# Supplementary material for: Are carbon emissions trading and green financial instruments synergistic? -Comprehensive quantitative research based on content analysis
Source: PLoS One. 2024 Mar 7;19(3):e0298601. doi: 10.1371/journal.pone.0298601 (PMC10919601; doi:10.1371/journal.pone.0298601)
Supplement: S1 Data — (ZIP) [file pone.0298601.s001.zip › Data/地级市Z_F工作报告.zip!/║╙▒▒/└╚╖╗╩╨/└┤╫╘╣1⁄2╓┌║┼í╛┬φ┐╦╩2╛▌═°í┐_2012.docx]

各位代表：

　　现在，我代表廊坊市人民政府向大会作工作报告，请予审议，并请市政协委员和列席会议的同志提出意见。

　　一、2011年工作回顾

　　2011年是“十二五”起始之年。面对国内外错综复杂的形势，我们在省委、省政府和市委的坚强领导下，坚持以科学发展为主题，以加快转变经济发展方式为主线，着力实施精良管理，积极推进高端发展，较好地完成了市五届人大四次会议确定的各项任务，实现了“十二五”良好开局。

　　经济发展再上新台阶。预计全市地区生产总值完成1650亿元，同比增长11.5%。全部财政收入完成251亿元，增长28.6%；其中地方一般预算收入完成140亿元，增长32.5%，占全部财政收入比重达到55.8%，高出全省平均水平8.9个百分点。全社会固定资产投资完成1089亿元，增长30.7%。规模以上工业增加值完成664亿元，增长16.7%。社会消费品零售总额486.3亿元，增长17.6%。全市经济实现了高质量、有内涵的增长。

　　产业结构得到新提升。以建立现代产业体系为方向，着力构筑高端定位、创新引领、集聚发展的产业新格局。电子信息成为发展最快的新兴支柱产业，实现利税和工业增加值均居全省第一位。休闲商务、金融服务、健康医疗等现代服务业迅猛发展，国寿生态健康城、鲁能生态旅游度假区、霸州国际温泉公园等项目进展顺利，梦廊坊文化产业园、北方金融后台服务基地等项目加快实施，村镇银行和农村商业银行快速发展，光大、交通、兴业银行落户。网络经济、总部经济、创新经济势头良好，润泽国际信息港、中国移动北区中心、中国联通华北基地、能源生态城项目加快建设，中太建设集团总部跨入中国企业500强。现代农业蓬勃发展，恒都美业、海泽田等4个现代农业园区初具规模，金丰农科园、天圆山庄等一批综合生态农庄迅速兴起。园区平台进一步提升，10个省级工业聚集区、6个省级新兴产业示范区、4个省级开发区获批，燕郊国家级高新区揭牌。项目中心建成使用，电子管理台账启动运行，争取重点项目新增用地指标再创新高。节能减排完成省下达任务，实施208项减排工程，达到年度控制要求；万元GDP能耗下降3.66%，继续保持全省最低。

　　城乡面貌实现新改观。城市建设强力推进，万达广场城市综合体建成营业，廊和坊金融街开业在即，京沪高铁站建成使用，苏宁广场、广阳CBD、文化中心、自然公园西扩等项目加快实施。市区新增绿化面积32.4万平方米，绿化覆盖率达到46.5%，人均公共绿地面积13平方米，空气质量一级天数123天，首次突破百天。编制完成城市总体规划纲要、中心城市空间发展战略规划和廊北新区概念性总体规划。新一轮土地利用总体规划争取省定向追加用地38平方公里，位居全省首位。城市网格化管理覆盖面继续拓展，数字城管向县域延伸。重大基础设施建设实现突破，廊沧高速建成通车，密涿支线高速具备通车条件，京台高速开工建设，南水北调廊涿干渠开工建设，广阳水库获批立项，调引9200万立方米黄河水入廊，文安县城新防洪圈主体工程全面启动，14项110千伏及以上输变电工程投产。新农村建设成果显著，建成112个省级示范村，1.5万户农民迁入新居，改建农村公路360公里。全市造林17.7万亩，建设了三河森林公园、霸州生态公园等一批精品工程，森林覆盖率达到24%。

　　农业农村经济取得新进展。粮油、蔬菜、畜牧、林果产业规模化和标准化水平提高，粮食生产连续八年增产，肉、蛋、奶、菜等农产品产量全面增长，建成40个千亩蔬菜标准园，新增节水灌溉面积13万亩、设施农业15万亩。农产品加工业进一步发展，农业产业化经营率达到65%，新增市级龙头企业42家，国家级龙头达到5家。三河农业高新区成为国家农业产业化示范基地，固安成为国家农业综合开发示范区。

　　改革开放迈出新步伐。重点领域改革深入推进，基层医药卫生体制综合改革全面完成，香河医改经验在全省推广。市区公交实施运营体制改革，实现公车公营，投入200部新型智能公交车，公交面貌焕然一新，社会满意度明显提升。明珠集团整体改制顺利推进，华夏幸福基业、梅花生物科技成功上市，新增5件中国驰名商标。信用建设走在全国前列，荣获“信用体系建设杰出贡献奖”。民营经济稳步发展，营业收入增长35.6%，新增民营企业4224家；亿元以上民营担保机构达到21家，居全省第二。对外开放取得新成绩，全市实际利用外资5.8亿美元，进出口总值52.9亿美元。成功举办北京·廊坊周，与央企、京企达成投资意向29项、总投资210亿元。

　　民生保障得到新改善。城镇居民人均可支配收入22818元、农民人均纯收入8500元，分别增长12.6%和12%。城镇登记失业率控制在2%以内，继续保持全省领先。农村劳动力转移10.6万人次，城镇新增就业岗位4.6万个，在全省率先实现毕业生就业网上登记。城镇居民养老保险启动实施，城镇居民医疗保险制度实现全覆盖。新农合参合率达到94.7%，补助标准由每人120元提高到200元，住院补偿限额提高到6万元。在全省率先建立大病医疗救助基金和“一站式”即时结算服务体系，全年发放城乡医疗救助资金2147万元。城乡低保实现应保尽保，农村敬老院管理服务水平保持全省先进。社会救助体系进一步拓展，制定实施支出型贫困家庭救助制度，贫困大学生助学覆盖面达100%。建设8座农村集中供水水厂，解决20万人饮水安全问题。实施市区金桥、金光小区旧改工程，受益家庭4100多户。完成保障性住房建设任务22996套，占省定目标的116.7%。李克强副总理视察廊坊，对我市住房保障工作给予充分肯定。

　　社会事业展现新局面。科技支撑引领作用得到增强，新建4家科技企业孵化器、8家企业技术研发中心、2家省级工程中心，实现高新技术产业增加值254.5亿元，增长31.5%。中长期教育改革发展纲要制定实施，义务教育均衡发展成效明显，标准化学校建设加速推进，农村学校办学条件明显改善，中小学校舍安全得到教育部肯定，市卫生学校升建职业学院，首届中国农村教育论坛成功举办。文化信息资源共享工程城乡覆盖率达95%，实现所有乡镇都有文化站，廊坊被命名为中国龙凤文化之乡，霸州成为全国县级公共文化服务体系建设典型，大型历史剧《宋相吕端》进京演出并获中央媒体和社会各界高度评价，隆福寺项目进展顺利。新建改建县级医院5个、乡镇卫生院22个，90个乡镇卫生院和586个村卫生室全部实行基本药物零差率销售。民族团结进步工作取得新突破，大厂被授予“全国民族自治县科学发展示范县（旗）”。全民健身运动广泛开展，人口计生工作继续加强，安全生产和食品药品安全得到保障，社会治安和信访稳定不断强化，国防动员和双拥共建深入实施，民族宗教、妇女儿童、扶残助残、防震减灾、物价、档案、人防、气象等各项社会事业取得新成绩。

　　政府自身建设全面加强。始终坚持依法行政，认真听取各民主党派、人民团体和社会各界意见，科学决策、民主决策得到有效保障。自觉接受人大法律监督、政协民主监督和社会监督，办理人大代表建议、政协提案417件，按时办复率100%。深入开展创先争优活动，大力推进效能廊坊建设，行政许可事项削减至115项，在全省率先建设标准化行政服务平台，率先实施电子监察市县对接、同城审批。规范执法程序和行为，推行“三步式”柔性执法，不断强化督查考核与审计监督，严格落实党风廉政建设责任制，推行行政权利公开透明运行，政府工作的执行力和公信力有了新提高。

　　各位代表，过去的一年，我们面对复杂多变的形势，团结拼搏，共克时艰，取得了来之不易的成绩。这是省委、省政府和市委总揽全局、正确领导的结果，是全市人民同心同德、不懈奋斗的结果。在此，我代表廊坊市人民政府，向全市人民，向人大代表、政协委员，向各民主党派、工商联、人民团体和各界人士，向驻廊解放军、武警官兵、政法干警和中省直驻廊单位，向所有关心支持廊坊发展的海内外朋友，致以崇高的敬意和衷心的感谢！

　　在肯定成绩的同时，我们也清醒地看到，在我市经济社会快速发展中，工业化、城镇化、农村现代化“三化同步”还不够协调，产业结构、城乡结构、资源环境方面的一些深层次矛盾尚未根本解决。新兴产业发展虽快，但整体规模不够大，产业链条还不完整，园区档次、功能有待提高，城乡基础设施和公共服务能力还存在较大差距。特别是在部分干部的思想状态上，还存在自满自足意识，没有感到标兵渐远、追兵迫近的压力，缺乏应有的危机感和紧迫感。对于这些问题，我们要采取更加有效的措施，在今后工作中认真加以解决。

　　各位代表，回首过去的一年，我们携手奋进，共铸辉煌；展望新的一年，机遇在前，充满希望。我们一定倍加珍惜来之不易的基础和优势，倍加重视前进中的问题与困难，坚定不移地推进精良管理，追求高端发展，奋力开创经济社会发展的新局面！

　　二、2012年工作的总体要求和主要目标

　　今年是实施“十二五”规划承上启下的重要一年，我们将迎来党的十八大胜利召开，做好今年的政府工作意义重大。

　　省第八次党代会描绘了建设经济强省、和谐河北的宏伟蓝图，市第五次党代会明确了廊坊今后五年的奋斗目标、主要任务和重要举措，开启了精良管理、高端发展、加快实现“两个率先”的新征程，极大地激发了全市人民在新的起点上跨越前进的干劲和热情。综合研判国内外形势，我们既面对不少严峻挑战，也面临许多有利条件。从不利因素看，世界经济形势严峻复杂，增速放缓与物价上涨相互交织，经济发展面临的不确定因素增多。全国各地和周边地区竞相发展，省内各市相互赶超态势强劲，不进则退，慢进也退，我们面临更加激烈的区域竞争。从有利条件看，新一轮科技革命在发展中蕴育突破，国际范围内产业结构调整和转移加快推进，为我们培育壮大战略性新兴产业创造了条件。我国经济长期向好趋势没有改变，宏观调控更加注重转变发展方式，有利于发挥我市产业体系的比较优势。京津冀区域经济一体化和首都经济圈纳入国家“十二五”规划，北京实施东扩南拓战略，首都新机场将在京廊交界建设，环首都绿色经济圈建设加快推进，这些利好因素叠加形成的发展机遇前所未有，为经济社会发展注入了更为强劲的动力，标志着廊坊步入了历史性、跨越性、突破性发展的崭新阶段。特别是今年将召开党的十八大，必将极大地激发全市人民推进科学发展、促进社会和谐的积极性。

　　我们一定要准确把握宏观形势和政策取向，保持谨慎乐观态度，坚定率先发展信心，力求把困难和问题考虑得更充分一些，把各项措施准备得更周密一些，力求把发展的步子迈得更稳一些、更大一些，努力在全省发挥引导性、示范性、带动性作用。

　　今年政府工作的总体要求是：全面贯彻落实中央、省经济工作会议和市第五次党代会精神，秉持创新破难、遇艰愈奋、好中求快、稳中求进的工作方针，紧紧围绕“京津冀电子信息走廊、环渤海休闲商务中心”发展定位，实施精良管理，追求高端发展，推进增量存量同步调优，推进工业化、城镇化和农村现代化协调发展，着力稳增长、调结构、优环境、筑平台、惠民生、促和谐，以集中力量办大事、聚精会神抓落实的干事气魄和务实态度，努力实现廊坊经济社会更好更快更大发展，在“两个率先”的征程中迈出更加坚实的步伐。

　　全市经济社会发展的主要预期目标是：地区生产总值增长10%以上，全部财政收入和地方一般预算收入均增长18%以上，规模以上工业增加值增长15%以上，全社会固定资产投资增长20%以上，社会消费品零售总额增长17%以上，城镇居民人均可支配收入增长12%以上，农民人均纯收入增长11%以上，居民消费价格涨幅控制在4%左右，万元GDP能耗、化学需氧量、二氧化硫等节能减排指标完成省下达的年度目标任务。

　　实现今年的经济社会发展目标，关键是把握好以下几个方面：坚持速度规模与质量效益相统一，牢牢把握稳中求进的工作总基调，牢牢把握更好更快更大发展的总要求，选准突破点，培育增长点，在保证质量、提高效益、优化结构的前提下，能发展多快就发展多快。坚持城镇建设与农村发展相统一，充分发挥城镇化的引领作用，坚持以城促产、产城互融，推动以城带乡、以工促农，努力形成工业化、城镇化和农村现代化良性互动的发展格局。坚持存量做优与增量做强相统一，努力在存量挖潜和增量引导上下功夫，盘活现有企业、项目、园区的各类资源，充分释放发展潜能。提高资源配置效益，优化项目投资结构，努力形成新的效益增长点。坚持经济发展与社会建设相统一，在推进经济建设的同时，更加注重社会建设，着力提高群众生活品质，全面提升社会文明程度、社会管理水平和生态环境质量，促进经济社会和谐发展。

　　做好今年的政府工作，核心是把发展实体经济作为现实基础、把加快改革创新作为强大动力、把保障和改善民生作为根本目的，切实抓好以下着力点：一是千方百计稳增长，努力消除经济社会发展中的不平衡、不协调、不可持续因素，全力保持经济平稳较快发展和社会大局稳定，做到好中求快、稳中求进，打造河北率先发展的新高地。二是坚定不移调结构，不断优化投资结构，提升技术结构、产品结构、产业布局结构，着力在引进大项目、培育大企业上下功夫，在延伸产业链、强化集成优势上下功夫，在改造传统产业、增强创新驱动能力上下功夫，努力走出产业高端发展的新路子。三是坚持不懈优环境，积极营造优质高效的政务环境、公平公正的法治环境、健康有序的市场环境、诚实守信的人文环境、优美宜居的生态环境，形成干事创业、和谐稳定的环境新优势。四是全力以赴筑平台，加大基础设施、公共设施建设力度，完善提升教育、医疗、交通等配套功能，推进城镇和园区建设上水平。五是尽心竭力惠民生，牢固树立以人为本的执政理念，把人民群众的所想、所盼、所需，作为工作的明确方向和重要内容，着力解决好涉及群众利益的重点、难点、热点问题。六是凝心聚力促和谐，加强和创新社会管理，继续实施首都“护城河”工程，确保社会大局和谐稳定。

　　各位代表，今年的任务非常艰巨，面临的形势充满挑战。只要我们坚定创新破难的决心，抱定率先发展的信念，就一定能够创造新业绩，实现新突破！

　　三、2012年经济社会发展重点工作

　　按照今年经济社会发展的总体要求，坚持统筹、高端、依法的原则，突出重点，突破难点，着力做好八个方面的工作：

　　（一）加快产业结构转型升级。以调结构转方式和增强自主创新能力为重点，实施大企业引领、大项目支撑、集群式发展战略，加快构建具有国际竞争力的现代产业体系。

　　一是改造提升传统产业。坚持把改造传统产业作为转型升级的重要任务，深入开展对标行动，重点实施100项工业企业技改项目，提高企业技术装备水平，推进金属冶炼压延、食品加工、家具制造、保温建材等传统优势产业转型升级。推动产学研深度合作，依托北华航天、建筑机械研究院建立公共服务机构，为产品技术升级搭建平台。深入实施品牌战略，引导企业大力引进新技术、研发新产品，重点组织30家企业，争创省名牌、中国名牌等省级以上质量奖。

　　二是加快发展新兴产业。坚持把战略性新兴产业作为转型升级的主攻方向，培育壮大电子信息、装备制造、清洁能源、生物医药等产业，努力在规模化、集群化上迈出更大步伐。准确把握电子信息产业发展趋势，以现有龙头项目为依托，有针对性地引进建设一批具有国家资质的软件服务企业，促使软件服务业加快发展。加快推进汉能全球研发中心、中轻集团造纸装备、首钢装备制造基地等项目建设，大力发展整机和成套设备制造，培育一批高端装备制造领军企业和知名品牌。做大做强新奥能源生态城、煤基清洁能源、洁能国际太阳能等产业龙头，完善产业链条，打造清洁能源产业集群。推进生物医药、新材料、节能环保等产业聚集发展、壮大规模。

　　三是大力发展现代服务业。坚持把现代服务业作为转型升级的重要突破口，面向京津高端休闲商务需求，加快发展网络经济、总部经济、金融服务、文化创意等现代服务业。以构建中国云中心为目标，积极推进中国移动北区中心、中国联通华北基地等项目建设，力促网络经济形成规模。研究制定总部经济发展规划，以引进区域总部、营销总部、研发总部、金融总部为重点，积极促成奔驰汽车、通用电气、阿迪达斯等总部项目落户，加快总部经济聚集发展。推进中国北方金融后台服务基地、安邦保险燕郊后援中心、廊坊服务外包基地等项目，大力发展面向北京的金融后台服务产业。立足区位和产业优势，推进6个省物流产业聚集区和环首都现代物流园区建设，提速燕郊空港、物美集团、邮政仓储等重点物流项目建设，推进物流产业链条式、互动式发展。加快推进鲁能生态旅游度假区、汇福国际健康中心等健康休闲产业项目，努力建设京津高端人才休闲度假、创业居住的首选地。出台《廊坊市文化产业发展规划》，积极推进梦廊坊文化产业园建设，按照国家级标准，规划建设主题大剧院群、汉唐与西洋文化街、剧创中心等项目，努力争列国家级文化产业园区，打造京津地区文化旅游重要节点；加快推进国华影视基地、新闻出版产业园等项目建设，不断壮大文化创意产业。

　　四是增强自主创新能力。坚持把自主创新作为转型升级的关键环节，努力使自主创新成为廊坊的主流和特色，成为实现高端发展的第一推动力。继续加强创新载体建设，依托科技谷、清华科技园等创新基地，吸引各类科研院所、大中企业在我市建立研发中试机构。引导企业建立技术研发中心、重点实验室，支持园区、基地建立孵化器，新建5－10家市级企业技术研发中心，取得关键技术成果100项。积极培育北华航天、新奥研究院等一批技术转移机构，争取1－2家成为国家和省示范机构。组织实施一批重大技术攻关项目，引导科研机构、企业积极承担和参与重大科技专项。谋划建设廊坊科技园，借鉴中关村“异地共建、一区多园”建设模式，引进京津高端科研院所和创新团队，促进京津科技资源向我市流动。

　　五是强力推进节能减排。坚持把节能减排作为转型升级的重要举措，落实节能减排目标责任制，合理分解年度节能减排指标，强化督导奖惩和限批措施，确保完成年度任务。加强重点行业和重点单位监管，抓好新老“双三十”单位和42家重点用能企业节能降耗。探索发展天然气分布式能源，推动清洁能源的综合高效利用。实施200项重点减排工程，关停200家重污染小企业，监控200家重点企业，对违规排污实施多地区、多部门联防联治，严管重罚。节能减排牵涉全局长远，关系子孙后代，我们一定以更大的力度、更硬的措施，打好这场攻坚战。

　　（二）切实加强重点项目建设。坚持把项目建设作为稳增长、调结构的重要抓手，进一步加强项目建设和管理，力求实现更大突破。

　　一是加大项目建设力度。全年实施600个投资亿元以上项目，全力推动“四个一批”：加快建设一批，提速润泽信息港、航天振邦、中石油压缩机组维修中心、康师傅食品工业园等59个投资10亿元以上在建项目进度，力促尽快投产达效。新开工一批，对中建机械制造基地、数字微波通讯、国电廊坊热电联产、吉利霸州产业基地等20个投资10亿元以上计划开工项目，千方百计落实建设条件，促其尽快开工。谋划储备一批，抓住三河铂阳太阳能，以及与央企合作的一批洽谈项目，跟进做好前期工作，促其尽快落地；继续抓好大城煤田综合勘探项目，加快推进前期普查勘探工作，为达到建井条件奠定基础。存量做优一批，对已落地的实体项目进行全面梳理和重点支持，深挖富士康、香河第一城等项目的后续潜力，用足资源，做优做强。

　　二是加强项目建设管理。研究出台加强重点项目建设的政策措施，实行重点项目分级管理，市县两级集中力量建设一批符合产业政策、有利于调结构转方式的大项目好项目。用足用活省重点项目支持政策，积极争列省重点项目。加强项目用地管理，严格按照土地容积率、单位投资率和预期回报率的高低依次供地，最大限度地向优势产业集中、向重点项目集中。进一步完善项目考核评价办法及奖励机制，采取电子台账与拉练观摩相结合的方式，实行每季调度、半年拉练、年终考核，激发项目工作的积极性。继续实施市级领导分包重大项目责任制，从招商洽谈到落地建设、投产达效，一包到底。

　　三是优化项目投资结构。准确把握产业高端发展导向，瞄准总部经济、网络经济、创新经济上项目，全力实施专业招商，吸引一批规模大、科技含量高、具有支撑和引领作用的产业项目。着眼提高产业丰厚度和关联度，加强龙头项目引进，促进配套项目聚集，推动产业链向高端拓展。发挥政府投资的引领作用，引导资金更多地投向产业项目、技术改造、科技创新、生态环保、民生改善等领域。引导民间资本由传统产业向新兴产业转移，避免低水平重复建设。强化择商选资和高端发展意识，加强对项目质量的评价核查，切实把好土地、信贷、节能、环保、产业政策等审核关，决不让低质低效项目挤占宝贵的土地资源，为未来发展留足空间。

　　（三）提升园区建设水平。切实加强园区的建设管理，整合园区资源，促进园区建设整体上档升级，努力把廊坊的园区优势转化为发展优势。

　　一是明确定位。制定出台全市加强园区建设的实施意见，开展新一轮园区规划提升工程，进一步调整优化园区体系，明确园区发展定位与产业分工，引导各园区立足现有基础，发展特色产业，建设专业园区，提高入区项目的关联度，形成错位发展、优势互补的园区体系，避免同质化发展和无序竞争，力争实现一个园区做强一个主导产业、培育一个产业集群。

　　二是强化功能。按照做园区就是做城市的理念，高标准推进水、电、路、气、讯、污水处理等基础设施建设，完善餐饮购物、医疗教育、休闲娱乐等配套功能，增强产业聚集发展的吸附力和承载力。营造招才引智的政策环境，加快专业人才和管理人才引进。每个县（市、区）都要集中力量打造一个精品园区，成为高端发展、率先发展的先导区、示范区。

　　三是完善机制。进一步强化项目准入机制，提高项目门槛，着力引进投资规模大、科技含量高、综合效益好的项目，提高土地利用率和投入产出率，珍惜土地资源，预留发展空间。进一步强化项目退出机制，从清理整治低效闲置用地入手，对不履行合同、不按期开工、慢建久拖、达不到约定进度的项目，进行全面排查、依法清理，有效盘活批而未用的存量土地，实现园区土地资源集约利用、高效开发。

　　四是做强重点园区。提升廊坊开发区和燕郊高新区综合功能，充分发挥示范带动作用，建立利益分享机制，积极探索“一区多园”建设模式。着力推进廊坊新兴产业示范区、科技谷等重点园区建设，尽快启动建设中关村固安高新技术产业园、嘉民产业园等一批重点园区。紧紧抓住北京新机场建设、首都经济圈加快推进的机遇，举全市之力推进廊坊空港新区建设，尽快制定总体规划，完成前置审批手续，力争空港新区早日列入省级发展战略，努力建成引领区域产业结构调整、承接北京乃至世界高端产业转移的战略平台。

　　（四）加快新型城镇化进程。按照“生态、智能、休闲、商务”城市定位，不断完善城市功能，聚集优质产业，强化精细管理，推进城镇建设上水平。

　　一是完善城镇体系。结合新一轮城市总体规划修编，完善市域城镇体系规划，提升“一体两翼”城镇空间布局。推动中心城市由“小三点、大三点”向“小四点、大四点”组团结构拓展，推进主城区、固安、永清、廊坊新兴产业示范区同城化建设管理，拉大城市框架，拓展发展空间。充分发挥环首都绿色经济圈和京沪高铁、廊沧高速的拉动效应，完善提升北中南三大板块城镇体系，推动县城和重点镇扩容提质，统筹推进城镇、园区、产业和新农村建设，加快形成环北京、环天津两个城镇群，提高承接中心城市辐射和带动农村发展的能力。

　　二是实施重点工程。围绕构建“一轴一廊两环八大中心”城市主框架，谋划实施城建“双十工程”，进一步完善城市功能、提升城市品位。启动建设艺术大道贯通、商业中心二期、广阳CBD、光明商务岛等十大重点工程，重点打造“两区”、做靓“两线”，以艺术大道为轴线，整合提升会展、文化、体育等功能中心，建设高品位生态文化艺术新区；以京沪高铁廊坊站、交通中心、苏宁广场、龙河中心为节点，建设中央商务区；继续实施城区道路容貌整治和景观提升，推进西昌路、北凤道绿道绿廊建设，提升廊涿高速出口、京沪高速出口两条迎宾线道路景观水平。实施住房保障、市区交通、市容整治、环境质量等十大公共服务项目，重点推进园林绿化、供热站、污水处理工程，新建续建银河路、永兴路、常青路等8条道路改造工程，不断提升服务配套能力和便民水平。县城和重点镇实施“五个一”工程，建设一条精品街道、一个高标准公园、一个城市商业商务综合体、一个城市广场、一批公共设施，不断增强城镇综合承载力。

　　三是强化统筹推进。坚持把承载产业、方便民生作为城市规划建设的重要内容，促进城市总体规划、土地利用规划、产业发展规划和民生工程规划有机融合，配套建设医疗、教育、文体、出行、购物等功能节点，完善城市服务功能。坚持城市布局调整与产业结构调整有机结合，加强中心商务区、城市综合体项目建设，引进一批餐饮、休闲、娱乐知名品牌，满足市民和投资者高品位、多样化的服务需求。加快聚集科技研发、总部经济、商务休闲等高端服务业，提高城市发展的产业支撑和要素吸纳能力。坚持城乡统筹，推进城乡规划、基础设施、产业布局、生态环境、社会事业和管理体制一体化发展，让城乡居民共享发展成果、共享美好生活。

　　四是实施精细管理。以建设数字城市、智慧城市为目标，推进数字廊坊、数字规划、数字交通、数字城管各系统的整合对接，加快推进数字城管向县城延伸，基本实现全市域覆盖，提高城市管理的反应速度和效率。进一步探索城市管理权下放，实现管理重心下移；全面放开环卫保洁等作业市场，提升公用服务质量。加大各类施工管理力度，实现全过程管理和联动执法，保障周边环境整洁、交通畅通。加强土地执法，开展专项行动，坚决遏制土地违法违规行为。切实加大对违法超建、擅自改变规划用途项目的处罚力度，维护规划的严肃性，努力把我们的城市建设得更精美、管理得更精细、维护得更精致。

　　（五）毫不放松地抓好“三农”工作。坚持统筹城乡发展，全面落实强农惠农政策，进一步加快现代农业发展和新农村建设，促进农业增效、农民增收、农村增活力。

　　一是壮大特色优势产业。按照一产抓特色的要求，进一步壮大畜牧养殖、无公害蔬菜、优质粮油、林果花木四大主导产业。创建68个部省级标准化规模养殖示范场，加快建设5万亩无公害果品生产示范基地。推进7个蔬菜产业示范县建设，新增设施蔬菜种植面积6万亩以上，增强集约化育苗、标准化生产和品牌化销售能力，打响廊坊绿色健康蔬菜品牌。提高农业产业化水平，重点建设10个农业产业园区，发展100家农民专业合作示范社，谋划一批科技含量高、带动性强的产业龙头项目，农业产业化经营率达到66%。

　　二是做强城郊都市型农业。大力发展休闲农业，积极创建全国休闲农业与乡村旅游示范县，全力抓好永清乡村旅游开发、大厂北方国际万果园、香河农事体验观光园等项目。加快发展农庄型、基地型、订单型农业，充分发挥恒都美业、金丰农博园等龙头项目的带动效应，综合配置种养、加工、餐饮、休闲、体验等各种资源，构建产加销一体化产业链条。加快农村土地承包经营权流转，重点向现代农业龙头企业、产业集群、重大项目集中。强化都市农业的科技支撑，加快新技术研发、新品种引进，增强都市农业的品质和效益。

　　三是全力促进农民增收。加快发展农村二三产业，培育壮大农产品加工业，深入实施“万村千乡市场工程”，有效增加农民非农收入。加快农超对接，支持种养能手、返乡农民工创办实业，增加经营性收入。大力发展劳务经济，继续推进百万农民大培训、阳光工程和电波入户，提高农民转岗就业能力，增加工资性收入。真正把增加农民收入作为头等大事，千方百计让农民收入渠道多起来、钱袋子鼓起来、生活富起来。

　　四是强化农业基础设施建设。加大水、电、路、田建设力度，不断改善农村生产生活条件。新增节水灌溉面积10万亩，恢复改善20万亩，建成高标准农田2.1万亩，投产3项110千伏输变电工程，统筹推进县域间路网规划建设，改建提升农村公路120公里。

　　五是积极稳妥推进新民居建设。把新民居建设作为推进“三化同步”的有效抓手，注重园区建设和产业支撑，切实尊重农民意愿。启动建设200个省级新民居示范村，规划建设10个环首都绿色经济圈精品新民居示范点，争列20个省级新民居建设重点支持项目，努力推动新村新业良性互进，让农民的生活环境有新改善，生活质量有新提升。

　　（六）深入推进改革开放。把改革开放作为破解难题、增强动力和活力的根本途径，加大推进力度。

　　一是深化重点领域改革。加快国企改制步伐，推动明珠集团等改制企业建立健全现代企业制度。推进医药卫生体制改革，深入实施基本医疗保障制度、国家基本药物制度等五项改革措施。推进预算管理改革和投资体制改革，提高财政科学化、精细化管理水平，集中财力办大事。推进行政管理体制改革，积极配合省政府完成工商、质监行政管理机构下划工作。推进农村集体林权配套改革和水利体制机制改革，加快基层供销社体制改革。

　　二是扎实推进对外开放。实施招商引资攻坚年，全面加强与央企的战略合作，以“百家央企进河北”为契机，抓紧落实9个签约项目，逐项分解，抓好对接，争取项目早日落地，努力在引进央企上走在全省前列。精心组织好“5·18”经洽会、“9·26”农交会，提升展会品牌和影响力。继续举办北京·廊坊周，切实提高规模档次和招商实效。创新招商方式，以小团组招商和会展招商为主要形式，开展定点、定向的产业链攻坚招商，全力实施珠三角、美国硅谷等重点招商，筹备实施针对总部经济等高端产业的专项招商。

　　三是全面深入对接京津。按照吸纳承接、融合提升、重点推进、互利发展的要求，加快京津廊一体化进程。加速构筑环首都绿色经济圈，强力推动“四区六基地”建设，打造承接京津科技成果转化、资源要素转移的有效平台；整合北三县要素资源，着力推进廊北新区建设，努力建成环首都绿色经济圈的先行区和示范区。强化协调机制，主动与京津各部门加强联系，搭建京津廊行政协调机制常态工作平台。推进互联互通，加强与京津规划、交通等部门的联系沟通，促进与京津高速公路、地方公路、轨道交通协调对接，加快供电、供水、供气、通信等公用设施共建共享，逐步实现就业用工、医保、住房公积金等公共服务畅通互联。主动承接京津产业转移和城市功能疏解，积极引进京津资金、项目、人才和技术，促进园区共建和劳务合作，不断提升区域合作的广度和深度。

　　四是大力发展民营经济。认真落实促进民营经济发展的各项政策，深入推进全民创业，培育壮大各类市场主体，全市新增法人企业2000家。以政府主导、市场化运作模式，组建企业综合服务中心，建立健全创业辅导、人才培训、技术支持等服务平台，加大对中小微企业的扶持力度。谋划建设民营企业创业大厦，鼓励有条件的民营企业上市融资，积极推进并购重组、战略联盟、相互持股，实现强强联合，力争有更多企业进入全省乃至全国百强。加快实施企业家素质提升工程，大力培育“廊商精神”，打造“廊商团队”，造就一批具有战略眼光、勇于开拓进取的领军人才。全市民营经济营业收入力争达到7000亿元，同比增长20%。

　　（七）全面优化发展环境。坚持把环境作为第一竞争力，营造近悦远来、惠商利民的发展环境，与周边区域形成梯度差。

　　一是优化政务环境。开展省级行政绩效管理试点工作，深入推进效能廊坊建设。进一步清理削减行政审批事项，加强行政服务中心建设，构建市县乡村四级行政服务体系，实现市县电子监察系统网络对接、同城审批。进一步规范执法行为，推进执法标准化建设，对行政违规、办事三乱等顽疾明察暗访，严肃处理。对标全国先进，大力缩减办事程序和时间，让企业和群众更方便、更满意。

　　二是改善金融环境。加强对金融业的扶持，吸引域外金融机构来我市拓展业务，引导金融机构向县域延伸，提升服务地方经济发展的能力和水平。加强银企对接，扶持规范小额贷款公司和村镇银行发展，壮大各类担保机构，有效解决中小企业融资难问题。加强对非法集资的打击力度，积极防范化解金融风险。

　　三是提升信用环境。巩固国家级“信用城市”成果，理顺市县信用管理体制，完善社会信用监管平台，引进和培育信用服务机构，扩大信用评价、激励惩戒的应用范围，逐步建成全覆盖的社会信用综合管理体系。

　　四是巩固生态环境。以创建国家生态城市为抓手，不断加强污染防治和生态保护，切实提升生态环境优势。实施龙河节点景观、自然公园西扩、绿道绿廊建设等生态景观工程，加强环城水系后续管理，推动新开发项目实施雨水储蓄工程。重点实施环首都绿色经济圈生态造林、廊沧高速造林等六大绿化工程，全年造林28万亩，森林覆盖率达到25%。

　　五是强化基础设施环境。京台高速全线开工建设，年内完成控制性工程，廊沧高速北延连通廊霸线，积极推进密涿高速建设，超前谋划对接北京新机场路网项目，加快唐廊高速廊坊段前期工作进度，力争与天津同步启动。确保南水北调廊涿干渠今年建成，争取广阳水库早日开工，努力推动引黄调水常态化，有效缓解我市水资源短缺状况。

　　（八）加快推进以民生为重点的社会建设。坚持把增进群众福祉放在政府工作的首位，着力推进各项社会事业全面发展。

　　一是着力改善民生。今年，市级财政安排民生方面支出26.3亿元，同比增长27.1%。围绕医、食、住、行等群众关心的热点难点问题，集中力量抓好十项民生工程。

　　住房保障工程。加大中低收入人群和困难群体住房保障力度，努力实现居者有其屋。按照国家三个三分之一的标准，高质量完成1.68万套保障性住房建设任务，切实做到公开、公正、公平分配。市区谋划启动金悦小区、惠民小区二期等5个住房保障项目，加快推进幸福佳苑、北旺儒苑等7个在建项目。全面改善提升市区旧住宅小区水暖、外墙、路面等硬件设施及社区环境，创建两个集中改造3万平方米以上的精品示范小区，规范提高物业管理，提升市民居住环境质量。

　　饮水安全工程。把饮水安全当作关系民生的德政工程，以前所未有的力度加大投入、加速建设。今年投资2.55亿元，建设22座集中供水水厂，解决农村饮水不安全人口51.1万人，确保两年全部解决农村饮水不安全问题。

　　教育提升工程。加快市区学校建设，新建6所小学及附属幼儿园，扩建改造4所中小学，启动市一中、市四中迁建工程，有效缓解中小学班额过大问题。持续推进校舍安全工程，利用两年时间，实现全市校舍无危房，加强校车管理，全力消除安全隐患。加快义务教育学校标准化建设，力争全市20%以上的幼儿园、小学和初中实现标准化。大力提升办学水平，重点推进市一中创建全省一流高中、电子信息工程学校创建国家级示范校。着力提升全市教育均衡化水平，让更多学生享受优质教育资源，享有安全健康的教育环境。

　　公交改造工程。在去年投入200台新型智能公交车的基础上，今年再购置100台，开通开发区环线、广阳道横线、金光道横线和银河路纵线四条线路。加快公交场站建设，并纳入城市建设规划，启动市区客运西站、北站综合运输枢纽建设，尽快完成三个市区公交场站选址，力争年底前开工建设，进一步缓解交通拥堵问题。

　　全民社保工程。扩大社会保险覆盖面，城乡居民社会养老保险制度实现全覆盖，新农保参保率达到90%以上，新农合参合率稳定在95%以上，城镇职工医疗保险完成扩面5000人，参保率达到93%。全市新增社会化养老服务床位1600张，建设18个养老精品示范服务站。稳步提高社会保障标准，建立与经济发展相适应的社保标准增长机制。

　　就业促进工程。以高校毕业生就业、军转安置、失业人员再就业和农村劳动力转移为重点，不断扩大就业总量、提高就业质量。全市城镇登记失业率控制在2.5%以内，城镇新增就业4.5万人，失业人员再就业5500人，农村劳动力向非农产业转移6万人。

　　卫生强基工程。进一步完善政府主导、多元投入机制，积极引导社会资金参与医疗卫生事业，全面加强城乡医疗卫生服务体系建设。新改扩建8所二级以上医院，市第三人民医院投入使用，全面提升廊坊综合医疗服务能力。整合全市卫生急救资源，成立市120医疗急救指挥调度中心。建成8所政府举办的社区卫生服务中心，建设25个乡镇标准卫生院、222个村标准卫生室，实现每个乡镇都有标准卫生院、每个村都有标准卫生室。

　　文体惠民工程。出台城市公共文化设施专项规划，积极建设市群艺馆、美术馆、廊坊大剧院、龙河书院，谋划建设一批县级文化中心、特色博物馆、纪念馆和数字影院，全年完成159个农家书屋建设和3.8万场次电影放映。市区新建20个社区体育健身工程，每个县（市、区）建设1－2个样板体育健身工程和20个村级工程。

　　十小便民工程。计划投资2.2亿元，启动小街巷、小游园、小停车场、小信报箱、小型体育设施、便民公厕、便民市场、便民通道、便民综合体、社区卫生服务机构10个方面的整治提升和建设工程，着力解决好“一家一户办不到，千家万户都需要”的民生问题，让市民生活更舒心、更舒适。

　　食品药品安全工程。大力开展食品药品安全专项整治行动，加大对非法添加剂、瘦肉精、注水肉、地沟油和奶制品、酒类的整治力度。进一步健全常态化的食品药品安全监管机制，完成市、县两级政府食品安全委员会组建工作，对食品生产、加工、销售各环节实行全程监管，实施药品电子监管，完善食品药品安全三级联保责任制，确保群众饮食用药安全。

　　二是协调发展各项社会事业。加快构建自主创新体系，不断提升科技研发能力，深入推进国家可持续发展实验区建设，高新技术产业增加值增长30%以上。推进教育事业优先发展，切实提升学前教育、义务教育、高中阶段教育、高等职业教育水平。完善公共文化服务体系，全面推进农村文化信息资源共享工程，深入挖掘龙凤文化底蕴，制定弘扬龙凤文化的总体规划。推动群众性体育与竞技体育发展，完善全民健身服务体系，举办市第六届运动会，组织好2012年全国跆拳道锦标赛。健全公共卫生服务体系，高度重视卫生防疫，健全应急处置机制，强化疾病防控体系和妇幼卫生体系，全面提高基本公共卫生服务均等化和医疗服务质量，探索建立医患纠纷调处第三方仲裁机制。强化环保监管，确保环境安全。深入开展双拥共建活动，切实做好国防动员、人民防空和防灾救援工作，重视发展民族宗教、人口计生、妇女儿童、扶残助残、物价等各项事业，促进社会全面进步。

　　三是维护社会和谐稳定。发挥好首都“护城河”作用，为党的十八大胜利召开创造安全稳定的社会环境。强化社会管理创新，进一步建立健全社会管理综合治理体制机制，完善社会治安防控体系，强化预警和应急处置机制，加强对重点地区、重点部位、重点人员、重点物品的防范控制，努力打造京畿平安示范区。切实抓好信访稳定，构筑大信访工作格局，加强行政复议委员会建设，进一步完善大调解工作机制，深入开展社会矛盾和信访苗头隐患大排查、大调处活动，严格落实领导接访、下访和约访包案制度，努力把问题解决在基层、化解在萌芽。强化安全生产，落实安全责任，严格执法监察，加强重点行业和领域的监管，严防重特大事故发生。

　　各位代表，推进以民生为重点的社会建设，是我们义不容辞的责任。我们一定以更大的力度推进发展、改善民生、促进和谐，让廊坊人民生活得更加美好、更有幸福感！

　　四、全面提升推进科学发展的执行力

　　新机遇赋予新使命，新任务带来新挑战。实施精良管理，追求高端发展，加快推进“两个率先”，必须大力提升政府的执行力。

　　第一，努力提升科学决策的能力。健全决策机制，完善决策程序，对涉及全局、群众关注的重大事项，广泛征求意见，充分咨询论证。提高依法行政的自觉性和坚定性，严格按照法定权限和程序行使权利、履行职责。自觉接受人大法律监督和政协民主监督，认真听取民主党派、工商联、各人民团体和人民群众的意见建议，提高科学决策、民主决策的水平。

　　第二，努力提升统筹协调的能力。把握工作的系统性和整体性，善于从全局的角度思考问题、谋划工作，增强谋大事、抓统筹、促协调的本领。紧紧抓住牵动全局的重点工程、重大事项和关键环节，努力做到城市、农村、社会、民生、产业统筹谋划，同步推进。

　　第三，努力提升创新破难的能力。率先发展，贵在创新。坚持以创先争优活动为载体，以解放思想为核心，推进理念创新和工作创新，努力克服不思进取、甘于平庸、无所作为、自满自足的倾向，树立强烈的竞争意识，以更高的工作标准充分释放发展潜能，以更有效的创新举措实现工作突破。

　　第四，努力提升推动落实的能力。切实转变工作作风，深入基层，加强调研，集中力量办大事，聚精会神抓落实。切实强化实干意识，进一步精简会议和文件，改进文风会风，把更多的精力放到解决改革发展稳定的重大问题上，放在解决群众生产生活的紧迫问题上。切实强化责任意识，加强绩效管理，实行责任追究，做到事事有人管、件件有着落。

　　第五，努力提升廉洁持政的能力。始终牢记“两个务必”，坚决制止奢侈浪费，严格控制“三公”支出。全面落实党风廉政建设责任制，深入推进廉政风险防控管理，坚持从严治政、从严执纪。强化行政监察和审计监督，认真抓好工程建设、政府采购、资金使用等重点领域和重要环节的监管，严肃查处违法违纪案件。深入开展政风行风民主评议活动，加快推进政务公开，以为民、务实、清廉的好作风，造福全市人民。

　　各位代表，面对435万廊坊人民的期待，我们肩负重托、责任重大。让我们在省委、省政府和市委的坚强领导下，以更加开阔的视野、更加务实的作风、更加有力的措施，凝聚智慧，砥砺奋进，全面加快“两个率先”步伐，以优异成绩迎接党的十八大胜利召开！
